# Emergency Point-of-Care Blood Gas Analysis During Mass Gathering Events: Experiences of the Vienna City Marathon

**DOI:** 10.3390/jcm14072504

**Published:** 2025-04-07

**Authors:** Roman Brock, Mario Krammel, Andrea Kornfehl, Christoph Veigl, Benedikt Schnaubelt, Marco Neymayer, Daniel Grassmann, Andrea Zeiner, Patrick Aigner, Regina Gabriel, Susanne Drapalik, Sebastian Schnaubelt

**Affiliations:** 1 Department of Emergency Medicine, Medical University of Vienna, 1090 Vienna, Austria; 2 Emergency Medical Service Vienna, 1030 Vienna, Austria; 3 PULS–Austrian Cardiac Arrest Awareness Association, 1090 Vienna, Austria; 4 Arbeiter-Samariter-Bund Landesverband Wien, 1150 Vienna, Austria

**Keywords:** marathon, running race, medical contacts, blood gas, point of care, triage

## Abstract

**Background**: Long-distance running impacts many organ systems. Aside from musculoskeletal and cardiopulmonary events, the gastrointestinal and renal system as well as metabolic homeostasis and electrolyte balance can be affected. A respective medical support strategy enabling rapid diagnosis, triage, and treatment in the context of large sports events is thus of utmost importance. Incidents can be assessed and graded via point-of-care (POC) blood gas analysis (BGA). We thus aimed to evaluate the feasibility and benefits of its use during a large sports event. **Methods**: All documented patient contacts during the race of the Vienna City Marathon (VCM) 2023 were retrospectively assessed. Additionally, the BGAs conducted in all patients requiring intravenous access were analyzed. Data are presented in a descriptive manner. **Results**: There were 39,871 participants at the VCM 2023. Of these, 277 (0.7%) required medical support, localized most commonly in the finishing area of the race (n = 239, 86% of all incidents). Fifty-eight (20.9%) patients had to be hospitalized. The most frequent chief complaints were syncope or collapse (24.9%), followed by general pain (20.6%) and trauma (14.8%). Five patients (1.8%) suffered from seizures, and one experienced (0.4%) from spontaneous pneumothorax. Thirty-one patients (11.2%) received venous blood gas analyses, showing mean creatinine levels of 1.82 (±0.517) mg/dL, mean lactate concentrations of 6.03 (±4.5) mmol/L, mean pH of 7.42 (±0.0721), and a mean base excess of −0.72 (±3.72) mmol/L. No cases of hyponatremia occurred in the documented samples. In eight cases (25.8%), sodium concentrations were above 145 mmol/L, with a maximum of 149 mmol/L. No cardiac arrests occurred. **Conclusions**: The physical exertion during the assessed long-distance running race resulted in numerous contacts with the medical support teams. The use of POC BGA at a large-scale marathon event was shown to be easy and feasible, allowing for more extensive diagnostics on-site. It can be integrated into a medical support strategy and might be beneficial for decision-making regarding patient triage, treatment, hospitalization, or patient discharge.

## 1. Introduction

Running long distances presents a tough challenge for the human body. Not only are the musculoskeletal and cardiopulmonary systems stressed, but the physical activity can also cause problems in the gastrointestinal tract as well as renal, metabolic, and electrolyte disturbances [1,2,3]. Running causes physical stress for the muscles due to acceleration and deceleration and can result in physical damage, possibly leading to an increase in creatine kinase and rhabdomyolysis [1,4,5,6].

Furthermore, the physical activity demands higher cardiac performance, which might unveil unknown or congenital cardiac conditions such as coronary artery anomalies or disease or arrhythmogenic right ventricular cardiomyopathy [1,2,7,8]. This, in turn, can lead to cardiac injury or dysfunction [9,10,11,12] and ultimately to cardiac arrest [8,13,14,15,16]. The most prevalent underlying diseases in young athletes suffering from sudden cardiac death seem to be hypertrophic cardiomyopathy or anomalous coronary arteries [17]. The risk of sudden cardiac death during running a marathon is estimated as 0.35 to 1 per 215,000 h of running [18,19] or 2.6 per 100,000 finishers [8].

Not only are the heart and muscles affected by vigorous activity, endurance running also has an impact on the gastrointestinal system. Because of the higher oxygen demand, blood flow is redirected towards the active muscles, causing a relative reduction in intestinal perfusion, which may lead to a wide range of symptoms from cramping to ischemia and has been compared to shock states [1,20,21,22]. Gastrointestinal bleeding has also been reported, as a consequence of intestinal ischemia [23], and another underlying mechanism of gastrointestinal symptoms is mechanical trauma [24]. As a more exotic adverse effect of running a marathon, an exercise-associated pancreatitis has been reported [25]. The same mechanism of blood flow redirection, accompanied by dehydration, might also lead to renal impairment and even hematuria and acute renal failure persisting up to three days after the marathon [1,26]. Pulmonary problems in marathon athletes include bronchospasms induced by exercise, as well as pulmonary edema of non-cardiogenic cause [1] (for example, caused by hyponatremia [27,28]). The most important disturbance in metabolism is hyponatremia, most likely caused by sweating, dilution by fluid intake, or hormone imbalances. Hyponatremia might lead to a wide range of symptoms from dizziness to seizures, pulmonary edema, coma, or death [1,27,28]. One of the leading reasons for athletes needing medical support in the context of marathons is exercise-associated collapse [29], which may be attributed to dehydration, hyponatremia, or temperature dysregulation in terms of hypo- or hyperthermia [1]. Maximum oxygen uptake is known to be dependent on the training state of an athlete, and a correlation to the marathon time has been shown [30]. With increasing exercise intensity above an individual level, which has been shown to correlate highly with running times at marathon races, lactate begins to accumulate because of increased lactate production due to limited oxygen supply or limited lactate removal [31].

To account for all these possibilities of medical issues, a profound planning of medical support around a marathon race including staffing, communication, transportation, and medical tents is necessary to ensure rapid diagnostics and treatment in the case of emergencies [32]. Additionally, environmental conditions need to be taken into consideration [32,33].

Many of the mentioned serious medical issues associated with a marathon could be assessed via point-of-care (POC) blood gas analysis (BGA). POC BGA has in recent years been introduced as a routine diagnostic option in the physician-based emergency medical system (EMS) of the city of Vienna. We thus aimed to evaluate the feasibility of its use during the city’s annual largest marathon event, as well as to assess potential clinical benefits and diagnoses resulting from its use.

## 2. Materials and Methods

### 2.1. Study Aim

The aim of the present study was the retrospective exploration of patient contacts, medical interventions, and POC BGA in participants of the Vienna City Marathon (VCM) 2023 for a respective descriptive overview and evaluation of POC BGA feasibility.

### 2.2. Study Setting

**Figure 1:**
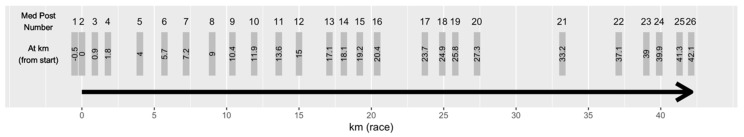
Distribution of stationary medical support posts (=Med Post). Km = kilometer.

The Vienna City Marathon (VCM) is an annual event taking place in Vienna, Austria. In 2023, the event took place on the 23rd of April, with a total of 39,871 participants. It consisted of three different competitions including marathon (42 km), a half-marathon (21 km), and a relay race. Medical support was handled by the Arbeiter-Samariter-Bund Vienna, one of the additional EMS organizations supporting the main EMS Vienna. The medical support staff consisted of 250 paramedics and eight emergency physicians operating in tents at various medical support posts, 35 ambulances, four emergency physician vehicles, and special units including bikes, Segways, and quads. The marathon had 26 stationary medical support posts along the way, with the main field ambulance situated near the finish line. Figure 1 depicts the distribution of medical support posts along the race. Mostly, marathon participants presented themselves to respective posts for short-duration ambulatory care, but, if needed, patients could be retrieved from anywhere at or near the race via support vehicles (ambulances, quads, bikes, and rangers) and brought to a nearby post. If longer-duration care or observation was needed, patients could stay at a post. However, if a definitive ambulatory care was not foreseeable, patients were transported to local hospitals. Of course, critical cases were treated with basic and advanced life support and hospitalized without further delay, similar to regular EMS missions. 

### 2.3. Data Acquisition and Analysis

All patient contacts and interventions during the marathon had routinely been documented, and, in 2023, POC BGA was for the first time introduced in routine care at medical support posts as a field test. BGA was conducted automatically if intravenous access was required at the discretion of the treating paramedic or physician. Blood samples were evaluated with one of four Epoc^®^ devices (Siemens Healthineers, Siemens Healthcare Diagnostics GmbH, Forchheim, Germany) [34]. Data were then retrospectively extracted from the available handwritten and electronic documentation sheets and POC BGA reports. No follow-ups were collected. The statistical analysis was performed with R 4.4.1 (R Foundation for Statistical Computing. Vienna, Austria. 2024). Numerical data are reported as means and standard deviations (SDs), as well as medians and interquartile ranges (IQRs). Categorical data are reported in absolute and relative numbers. *p*-values below 0.05 were considered statistically significant.

## 3. Results

**Figure 2:**
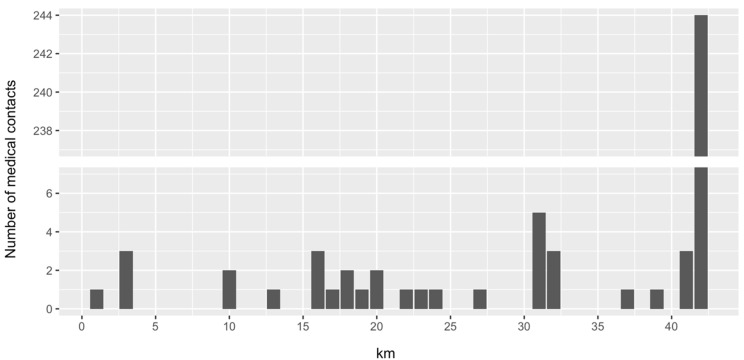
Medical contacts by elapsed distance (277 contacts in total).

In total, 39,871 people participated in the VCM 2023. A total of 277 (0.7%) incidents requiring support by medical personnel were reported, and patients were around 36 (±14.6) years old. Figure 2 shows the number of medical contacts during the race, stratified by elapsed kilometers. The vast majority of medical encounters were located at the main ambulance site in the finishing area (n = 239, 86% of all incidents). Of these, 58 (20.9% of all incidents) patients could not be discharged home on-site but had to be hospitalized instead. The most frequent chief complaints were syncope or collapse (n = 69, 24.9%), followed by pain (n = 57, 20.6% of all incidents; e.g., joint pain, muscle pain, and muscle cramps) and trauma (n = 41, 14.8%). Five patients (1.8%) suffered from seizures, and one (0.4%) suffered from spontaneous pneumothorax. No cardiac arrest was reported. In previous years of the VCM, however, cardiac arrests did take place. Table 1 shows an overview of the chief complaints. 

In 31 patients (11.2%), venous access was required. All these patients received a venous BGA during this field test, irrespective of their chief complaints. At the time of presentation, these patients had a mean creatinine level of 1.82 (±0.517) mg/dL and a mean lactate concentration of 6.03 (±4.5) mmol/L. Mean pH was 7.42 (±0.0721) and the mean base excess −0.72 (±3.72) mmol/L.

Patients requiring medical support who had aimed for a running distance of 42 km (n = 12) had significantly lower venous lactate levels compared to patients who had aimed for a running distance of 21 km (n = 7): 11.1 (±3.63) mmol/L vs. 3.26 (±1.37) mmol/L; *p* < 0.001. This was not the case for creatinine levels (1.90 (±0.386) mg/dL vs. 1.86 (±0.541) mg/dL; *p* = 0.86). In eleven cases (34.4%), the desired running distance could not be assessed from the records.

No cases of hyponatremia occurred in the documented samples. In eight cases (25.8%), sodium concentrations were above 145 mmol/L, with a maximum of 149 mmol/L. POC BGA results are summarized in Table 2. Of these 31 patients, eight (25.8%) had to be hospitalized (BGA measurements are summarized in Table 3).

Regarding POC feasibility, operators were able to conduct analyses successfully after a short verbal introduction. We only encountered thermal problems caused by direct sunlight, which can easily be avoided in the future. No other difficulties were encountered. The devices were redistributed on demand during the race without problems.

## 4. Discussion

The present study showed that POC BGA for routine use during medical support for mass gathering events (I) is feasible in the context of a large marathon race, (II) yields fast and valuable information for further decision-making regarding triage, treatment, and hospitalization, and (III) can be easily integrated into an already-existing well-designed medical support strategy. 

Regarding feasibility, the POC BGA device can be used productively after a short introduction and little to no training by specialist staff such as paramedics, nurses, or medical students. Prehospital BGA has already been described as feasible [35], and we have now shown that even in the case of a mass running event, the devices were able to keep up with the workload and produced fast results within minutes. Before starting a new analysis, the devices need an initialization period of approximately two minutes. The only issue encountered that needed special consideration was the environmental conditions, more specifically, heat by direct sunlight, which caused device failures and should be avoided. Using the devices in medical tents at mass gatherings can be a solution. Also, the devices are lightweight and small. Therefore, it was effortlessly possible to transport them from one site to another on demand. Additionally, the device runs on a battery, which sustained the whole race without the need of recharging.

A fast overview of respiration, electrolytes, metabolism, and renal function can be extremely useful in a low-resource setting with sparse diagnostic possibilities such as mass gatherings. In addition to aiding triage decisions, we suggest an increased efficacy regarding treatment and discharge or hospitalization in accordance with previous work by Pedersen et al. [36] and Mikkelsen et al. [37], who described the feasibility and benefits (especially the possibility of faster diagnosis and specific treatment) of an ABL-90 (Radiometer, Denmark) blood gas analyzer in a Mobile Emergency Care Unit (MECU) in Odense, Denmark, in the preclinical setting. Rief et al. analyzed 15 studies from Austria, Finland, Germany, Spain, the Netherlands, Denmark, Switzerland, and the USA, describing the prehospital use of BGA in ground-based as well as helicopter-based emergency medical services. Of these, 11 studies analyzed arterial samples, and five analyzed venous samples. pH, BE, pO_2_, and pCO_2_ were the parameters most commonly assessed. In total, they not only reported that a prehospital use was feasible and straightforward but also highlighted the diagnostic potential and prediction possibility of outcomes, especially in critically ill, cardiac arrest, or major trauma patients [35]. In Vienna, Austria, BGA is used in pre- and intrahospital settings routinely to complement other diagnostics as vital signs or ECGs to allow for faster and more precise initial patient management and monitoring. Also, during in- and out-of-hospital cardiac arrest, BGA is routinely used to rule out hyperkalemia and assess the metabolic condition (e.g., pH and lactate). Additionally, prehospital ultrasound is routinely utilized as a diagnostic support tool, but it is highly dependent on the skills and experience of the examiner and requires respective training [38].

Regarding changes in laboratory or BGA parameters in marathon runners, scarce data are available: Fitzpatrick et al. analyzed POC BGA parameters including electrolytes and creatinine in collapsed marathon runners at the Brighton Marathon between 2010 and 2019 in comparison to a non-collapse control group of 80 runners. They reported a significant increase in creatinine levels from pre to post-race in the control group and even higher values in the collapsed group post-race (with the lack of a baseline measurement in this group due to study design). Twenty-four hours after the race, creatinine values returned to the normal range. Additionally, they also showed a significant increase in urea and a decline in sodium concentrations in the control group [39].

Siegel et al. performed similar analysis in collapsed runners at the Boston marathon in 2006 and 2007. Aside from electrolytes, lactate, and BUN, they also, among others, analyzed troponin-T and NT-proBNP. They report an incidence of 13% for hypernatremia and 5% for hyponatremia, as well as an increase in BUN in 49% and lactate in 95% of runners. In collapsed runners, they found an elevation of troponin T above the respective threshold for myocardial necrosis in 8%. Only 5% of collapsed runners showed a modest increase in NT-proBNP [40]. Kratz et al. also analyzed the effects of a marathon on numerous laboratory parameters pre- and post-race, including creatinine and urea, and were also able to show a significant increase. The decline in sodium levels immediately post-race was not significant [41]. Using venous blood from finger sticks, Mohseni et al. analyzed sodium, potassium, renal function, and hemoconcentration in marathon and half-marathon runners pre and post-race and showed significant increases in sodium, creatinine, and urea [42]. Similar results, more specifically an increase in sodium, potassium, BUN, and creatinine, were presented by Nelson et al. in marathon runners at the 1987 Pittsburgh Marathon [43]. Kidney injury was specifically assessed by Atkins et al. via blood (post-race only) and urinary analysis (pre- and post-race) in participants of the Boston marathon. They showed an increase in urine specific gravity and urine creatinine and, furthermore, a normalization of serum creatinine within 24 h post-race [44].

Exercise associated collapse in the context of a half-marathon was also studied by Lüning et al. They analyzed incidences of severe collapse over a period of five years (2013–2017) at the Gothenburg’s half marathon. Aside from clinical data and vital signs, venous blood gases were collected as well in case electrolyte disturbances or hypoglycemia had to be ruled out. The reported incidence of collapse was 1.53 per 1000 starters, and further findings were acidosis in 38%, elevated lactate levels in 93%, and hypernatremia in 63% of the samples [45]. Of note, the values by Kratz et al., Nelson et al., and Atkins et al. were not measured by POC devices.

Regarding the measured values in our study, an interesting trend to drastically higher lactate concentrations in runners aiming for the half-marathon compared to runners aiming for the marathon distance was visible, possibly reflecting a poorer training state or running strategy. Measured creatinine levels were higher when compared to Fitzpatrick et al. [39], Kratz et al. [41], Mohseni et al. [42], and Nelson et al. [43] but lower compared to the data of Atkins et al. [44]. Sodium concentrations were comparable [39,40,41,42,43,46]. The observed lactate levels were higher compared to the analysis by Siegel et al. but slightly lower compared to Lüning et al. [40,45]. The studies mentioned above primarily focused on the description and comparison of affected laboratory parameters, but, aside from follow-ups, no direct interventions were initiated. With the inclusion of POC BGA measurements in an established medical support strategy, it would be possible to react upon pathological markers, guide therapies, and uncover more profound but possibly reversible problems especially in collapsed runners or ultimately in cardiac arrests.

In summary, the question arises if POC BGA devices should be used routinely at events such as the VCM. It must, however, be noted that this field test as a proof-of-concept study did not undergo economic calculations. It is most likely not target-oriented to perform a blood gas analysis on each and every patient but rather by specific selection criteria, which can only be hypothesized at this point and would need larger studies for evaluation. In our opinion, especially three types of patients might benefit from a POC BGA at sports mass gatherings:

- Patients in (semi-)critical condition to further assess the severity of impairment and to establish a basis for an early decision to hospitalize.
- Patients with multiple or profound medical conditions to create a more complete picture of the comorbid patient and aid further treatment.
- As decision support for early discharge on-site without further workup.

Aside from practical case selection criteria, additional factors must in the future be taken into account, such as optimal device count, localization, and potentially telemedical evaluation as proposed for various other sensors [47] or devices [37]. To address these knowledge gaps, larger prospective trials are necessary.

## 5. Limitations

Limitations of the present study primarily arise from the retrospective nature and the low number of patients. Further, the lack of follow-ups limits the assessment of the effective benefits of the utilization of BGA. Additionally, BGA was only performed at the day of the race at the time of medical support and at the discretion of the medical staff, lacking a standardized indication and the ability to assess trends in the measured values. Furthermore, no baseline data before the start of the race are available. The study is based on the organizational conditions and operating procedures of the Viennese emergency system. Therefore, the applicability to other systems must be assessed carefully. 

## 6. Conclusions

Long-distance running races such as the Vienna City Marathon present a stressful challenge to the human body, and participants may require immediate first aid or develop medical conditions lasting for several days after the race. Medical support around these mass gathering events, especially those with thousands of participants, is a huge undertaking and needs profound planning and staffing. POC BGA might be beneficial for decision-making regarding patient treatment, hospitalization, or discharge. Prospective studies are necessary to comprehensively assess the benefits of POC BGA during large events and the associations with clinical outcomes and treatment times.

## Figures and Tables

**Table 1:** Overview of chief complaints stratified by hospitalization.

	Hospitalization			
Chief Complaint	No (N = 219)	Yes (N = 58)	Overall (N = 277)	*p*-Value
Tachycardia	2 (0.9%)	1 (1.7%)	3 (1.1%)	0.507
Hypoglycemia	0 (0%)	1 (1.7%)	1 (0.4%)	0.209
Seizure	2 (0.9%)	3 (5.2%)	5 (1.8%)	0.064
Pain	54 (24.7%)	3 (5.2%)	57 (20.6%)	<0.001
Weakness	32 (14.6%)	7 (12.1%)	39 (14.1%)	0.832
Vertigo	8 (3.7%)	1 (1.7%)	9 (3.2%)	0.69
Trauma	29 (13.2%)	12 (20.7%)	41 (14.8%)	0.21
Collapse	47 (21.5%)	22 (37.9%)	69 (24.9%)	0.016
Angina pectoris	2 (0.9%)	1 (1.7%)	3 (1.1%)	0.507
Nausea, emesis	14 (6.4%)	5 (8.6%)	19 (6.9%)	0.561
Pneumothorax	0 (0%)	1 (1.7%)	1 (0.4%)	0.209
Other	29 (13.2%)	1 (1.7%)	30 (10.8%)	0.008

**Table 2:** Overview of initial venous blood gas values. BE = base excess.

Venous Blood Gas Analysis Values	Aimed Distance				
	Half-Marathon (N = 7)	Marathon (N = 12)	Relay Race (N = 1)	Unknown (N = 11)	Overall (N = 31)
pH					
Mean (SD)	7.37 (0.0435)	7.42 (0.0669)	7.40	7.46 (0.0773)	7.42 (0.0721)
Median (Q1, Q3)	7.38 (7.35, 7.39)	7.42 (7.38, 7.45)	7.40 (7.40, 7.40)	7.45 (7.43, 7.50)	7.43 (7.38, 7.46)
BE (mmol/L)					
Mean (SD)	−4.06 (2.60)	1.61 (2.70)	−4.9	−0.755 (3.66)	−0.719 (3.72)
Median (Q1, Q3)	−3.40 (−5.90, −2.30)	1.00 (0.0250, 3.50)	−4.90 (−4.90, −4.90)	0 (−1.05, 1.30)	−0.500 (−2.50, 1.05)
PO_2_ (mmHg)					
Mean (SD)	43.8 (17.2)	35.2 (17.6)	86.0	46.1 (11.9)	42.7 (17.6)
Median (Q1, Q3)	37.1 (32.9, 59.7)	33.4 (25.6, 39.2)	86.0 (86.0, 86.0)	48.6 (40.8, 53.9)	38.8 (31.6, 53.9)
PCO_2_ (mmHg)					
Mean (SD)	36.3 (8.60)	41.7 (10.7)	30.0	31.2 (5.45)	36.4 (9.43)
Median (Q1, Q3)	34.5 (29.6, 42.2)	41.2 (35.1, 48.1)	30.0 (30.0, 30.0)	30.6 (29.1, 34.0)	34.5 (30.4, 41.5)
Lactate (mmol/L)					
Mean (SD)	11.1 (3.63)	3.26 (1.37)	6.65	5.79 (4.89)	6.03 (4.50)
Median (Q1, Q3)	11.3 (9.62, 13.8)	2.67 (2.50, 4.23)	6.65 (6.65, 6.65)	5.56 (2.90, 5.88)	4.98 (2.75, 7.56)
Creatinine (mg/dL)					
Mean (SD)	1.90 (0.386)	1.86 (0.541)	1.46	1.76 (0.614)	1.82 (0.517)
Median (Q1, Q3)	1.76 (1.62, 2.11)	1.75 (1.59, 2.35)	1.46 (1.46, 1.46)	1.86 (1.36, 2.10)	1.77 (1.57, 2.24)
Na^+^ (mmol/L)					
Mean (SD)	145 (1.80)	141 (3.58)	145	143 (4.06)	143 (3.69)
Median (Q1, Q3)	145 (144, 147)	141 (140, 143)	145 (145, 145)	143 (141, 146)	143 (141, 146)
K^+^ (mmol/L)					
Mean (SD)	4.39 (0.279)	4.34 (0.605)	4.00	4.55 (0.327)	4.42 (0.448)
Median (Q1, Q3)	4.40 (4.20, 4.55)	4.10 (3.90, 4.83)	4.00 (4.00, 4.00)	4.50 (4.40, 4.80)	4.40 (4.10, 4.75)
Ca^2+^ (mmol/L)					
Mean (SD)	1.21 (0.0816)	1.19 (0.0648)	1.25	1.17 (0.0880)	1.19 (0.0759)
Median (Q1, Q3)	1.20 (1.17, 1.21)	1.21 (1.14, 1.23)	1.25 (1.25, 1.25)	1.17 (1.10, 1.24)	1.20 (1.14, 1.24)
Cl^−^ (mmol/L)					
Mean (SD)	107 (3.15)	104 (5.25)	110	107 (3.07)	106 (4.17)
Median (Q1, Q3)	106 (105, 109)	105 (102, 107)	110 (110, 110)	106 (105, 110)	106 (104, 110)

**Table 3:** Overview of BGA (blood gas analysis) values stratified by hospitalization. Data are presented as means and standard deviations. BE = base excess.

	Hospitalization			
BGA Values	No (N = 23)	Yes (N = 8)	Overall (N = 31)	*p*-Value
pH	7.42 (0.0462)	7.39 (0.104)	7.41 (0.0653)	0.32
BE (mmol/L)	−0.404 (3.13)	−1.27 (4.49)	−0.627 (3.47)	0.899
pO2 (mmHg)	43.6 (16.6)	36.1 (13.4)	41.6 (15.9)	0.597
pCO2 (mmHg)	37.3 (8.08)	40.3 (9.54)	38.1 (8.41)	0.506
Lactate (mmol/L)	5.53 (3.42)	6.72 (5.50)	5.84 (3.99)	0.272
Creatinine (mg/dL)	1.80 (0.480)	1.82 (0.636)	1.80 (0.514)	0.482
Na+ (mmol/L)	143 (3.81)	142 (3.63)	143 (3.73)	0.791
Ca2+ (mmol/L)	1.19 (0.0630)	1.21 (0.0683)	1.20 (0.0635)	0.924
K+ (mmol/L)	4.59 (0.588)	4.25 (0.347)	4.51 (0.552)	0.024
Cl− (mmol/L)	106 (4.24)	105 (3.51)	106 (4.04)	0.438

## Data Availability

The data underlying this article will be shared on reasonable request to the corresponding author, according to national law and organizational regulations.
